# Supplementary material for: Vestibular Compensation after Vestibular Dysfunction Induced by Arsanilic Acid in Mice
Source: Brain Sci. 2019 Nov 18;9(11):329. doi: 10.3390/brainsci9110329 (PMC6896078; doi:10.3390/brainsci9110329)
Supplement: Supplementary file 1 [file brainsci-09-00329-s001.pdf]

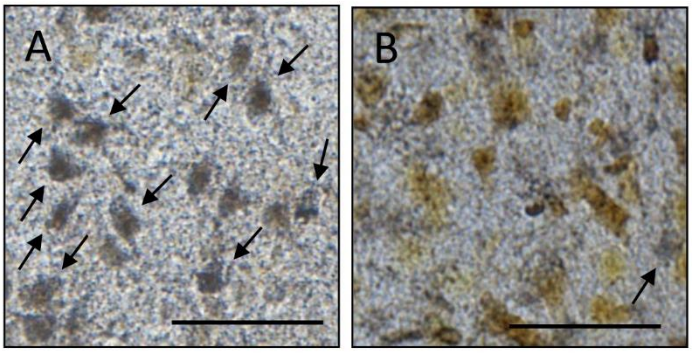

Figure S1: Silver staining of positive control tissues and the vestibular ganglion after unilateral labyrinthectomy with arsanilic acid in mice

(A) Positive control tissues provided with the staining kit. (B) Vestibular ganglion stained at 30 days after surgery.

Black-stained cells (arrows) can be seen in the positive control tissues (A). There are few black-stained cells in the vestibular ganglion (B).

Scale bar, 20 μm

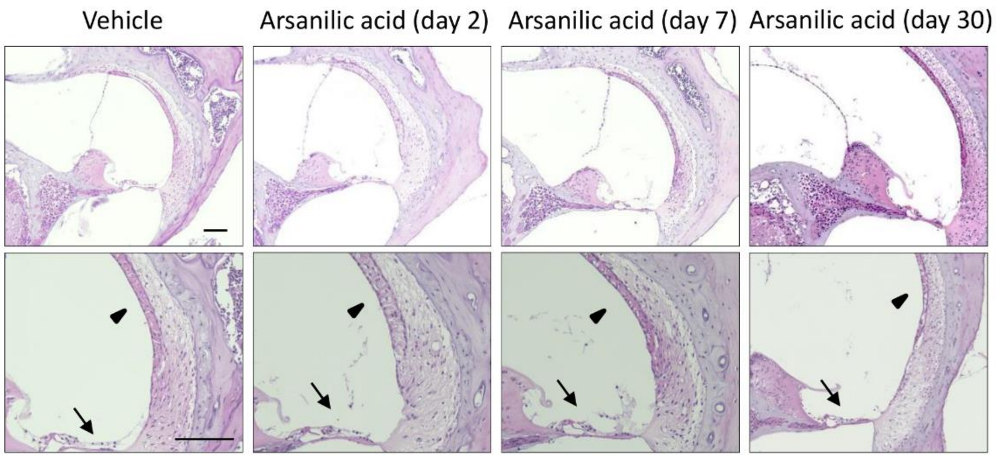

Figure S2: Histology of the cochlea after unilateral labyrinthectomy with arsanilic acid in mice (hematoxylin–eosin staining)

There is no significant damage to the hair cells in the cochlea (arrows) or to the stria vascularis (arrow heads).

Scale bar, 20 μm

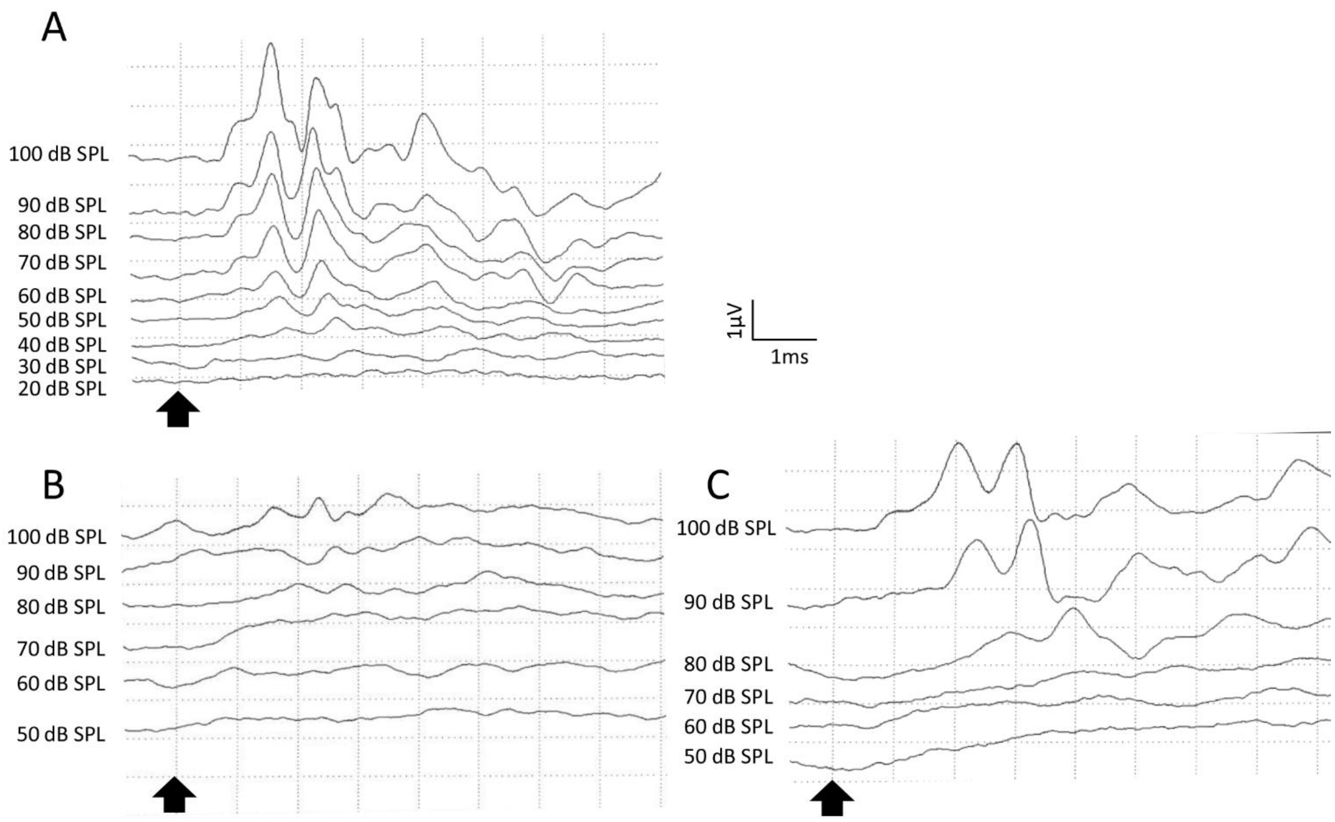

Figure S3: Auditory brain stem response (ABR) at 32kHz

A: intact group, B: arsanilic acid group, C: vehicle group

The thresholds of ABRs were successfully recorded among all groups: intact, arsanilic acid and vehicle group. The ABR threshold (wave I) in intact group was 30 dB. The thresholds were elevated by 30 – 40 dB on all conditions in both arsanilic acid and vehicle group, compared with intact group. There were no threshold shifts in the contralateral ears in both groups (data not shown).

Black arrows: triggers.

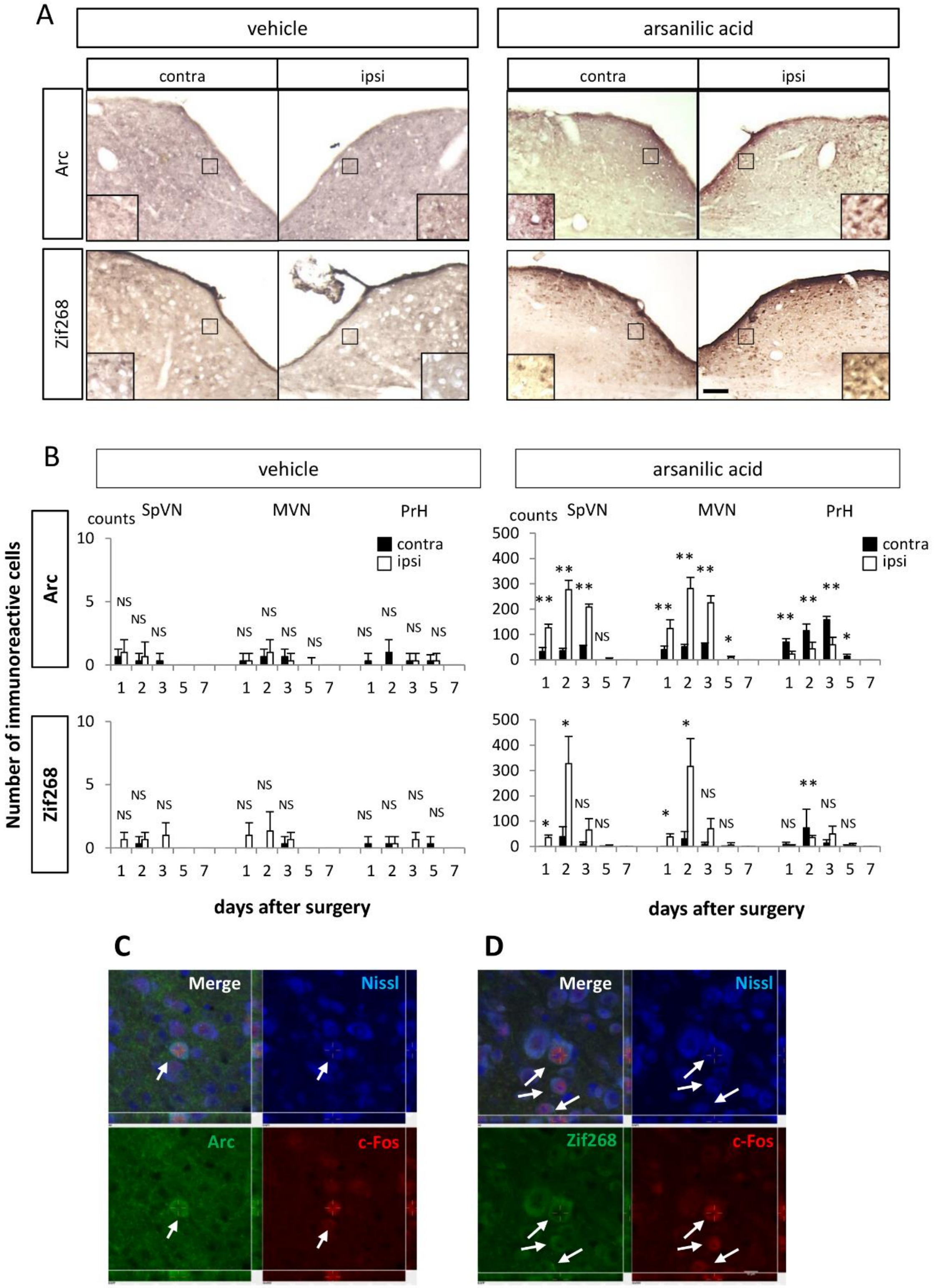

A
vehicle
arsanilic acid
contra
ipsi
Arc
Zif268
B
vehicle
arsanilic acid
Number of immunoreactive cells
counts
SpVN
MVN
PrH
contra
ipsi
NS
days after surgery
C
Merge
Nissl
Arc
c-Fos
D
Merge
Nissl
Zif268
c-Fos

Figure S4: Expression of Arc-IR and Zif268-IR cells in the vestibular nucleus and immunofluorescence double-labeling (c-Fos/Arc/Zif268)

(A) Expression of Arc-IR/Zif268-IR cells in the vestibular nucleus. (B) Number of Arc-IR/Zif268-IR cells. (C) Immunofluorescence double-labeling (c-Fos/Arc). (D) Immunofluorescence double-labeling (c-Fos/Zif268).

There are a few Arc-IR/Zif 268-IR cells in the SpVN, MVN, and PrHN in the vehicle (unilateral labyrinthectomy with phosphate buffer) group (A, B).

In the ipsilateral and contralateral SpVN/MVN/PrH in the arsanilic acid (unilateral labyrinthectomy with arsanilic acid) group, a substantial number of Arc-IR and Zif268-IR cells can be observed on day 1 after surgery, with a maximum increase in number at 2 days and a gradual decrease to zero by 7 days (A, B). This is similar to the pattern of expression of c-Fos-IR cells. The panels in C show the colocalization of c-Fos-IR (red) and Arc-IR (green) cells in the ipsilateral SpVN in the arsanilic acid group. The panels in D show the colocalization of c-Fos-IR (red) and Zif268-IR (green) cells in the ipsilateral MVN. The arrows indicate double-staining cells.

*$p < 0.05$, **$p < 0.01$

NS, not significant; one-way analysis of variance (ipsilateral vs. contralateral)
